# The Quality Improvement Challenge—How Nurses and Allied Health Professionals Can Solve the Knowing–Doing Gap in Enhanced Recovery after Surgery (ERAS)

**DOI:** 10.3390/medicina56120652

**Published:** 2020-11-27

**Authors:** Thomas W. Wainwright

**Affiliations:** 1 Orthopaedic Research Institute, Bournemouth University, 6th Floor, Executive Business Centre, 89 Holdenhurst Road, Bournemouth BH8 8EB, UK; twainwright@bournemouth.ac.uk; Tel.: +44-(0)1202-961656; 2 Physiotherapy Department, University Hospitals Dorset NHS Foundation Trust, Bournemouth BH7 7DW, UK

**Keywords:** enhanced recovery after surgery, quality improvement, surgery, orthopaedics

## Abstract

The English National Health Service (NHS), and all health services around the world, will continue to face economic and capacity challenges. Quality improvement (QI) interventions, such as Enhanced Recovery after Surgery (ERAS), that are proven to improve patient care and deliver operational benefits are therefore needed. However, widespread implementation remains a challenge. Implementation of ERAS within the NHS over the last 10 years is reviewed, with a focus on total hip arthroplasty (THA) and total knee arthroplasty (TKA). Difficulties with implementation are highlighted, and a recommendation for the future is presented. This perspective is novel in the ERAS literature, and centres around increasing the understanding of perioperative care teams on the need for utilising a recognised QI method (e.g., plan–do–study–act cycles, Lean, and Six Sigma) to implement ERAS protocols (which are a QI intervention) successfully. The importance of differentiating between a QI method and a QI intervention has value across all other ERAS surgical procedures.

## 1. Introduction

Health services around the world, including the English National Health Service (NHS), have faced economic and capacity challenges over the last 10 years, and these will remain and increase following the global COVID-19 pandemic. The ongoing reduction in resources and increasing demand for services, will provide an immense challenge to NHS organisations and staff. Quality improvement (QI) approaches may be used to improve the quality of patient care and save money, but their success is both dependent on the local context and how they are implemented [1,2]. Enhanced Recovery after Surgery (ERAS) (or enhanced recovery/fast-track) protocols are a QI intervention and are a multi-modal approach to care which has been shown to reduce mortality, morbidity, and length of stay (LOS) across a range of elective surgical procedures [3].

## 2. The History of ERAS Implementation within the NHS

ERAS protocols optimise the peri-operative pathway by minimising the surgical stress response to surgery by using and combining techniques, such as minimally invasive surgery, regional anaesthetic techniques, multi-modal opioid sparing pain management, early nutrition, effective fluid management and early mobilisation. ERAS protocols have been detailed in procedure specific evidence-based guidelines for a range of surgical procedures [3] and include recommendations for total hip arthroplasty (THA) and total knee arthroplasty (TKA) [4].

In England, the spread and adoption of ERAS was initially promoted over 10 years ago via a government led programme. The Department of Health (DOH) launched the Enhanced Recovery Partnership Programme (ERPP) in April 2009, which was a 2-year national improvement programme focused on surgical procedures involving the colorectal, urology, gynaecology, and orthopaedic (focusing on THA and TKA) specialties [5]. The ERPP aimed to reduce and address the wide variations in LOS found across common elective surgical procedures. ERAS protocols were an attractive intervention in order to improve clinical outcomes and increase the capacity required to meet the 18-week referral-to-treatment target. In year 1, the ERPP focussed on increasing awareness of ERAS through events, conferences, and producing supportive literature and online resources. In year 2, the ERPP focussed on spread, adoption and sustainability of ERAS, and amongst other activities produced a basic national ERAS database as well as encouraging regional support through the strategic health authorities.

There is a perception that ERAS strategies have been universally adopted in England; however, recently published data suggest that this is not a reality [6]. For some hospitals, ERAS protocols have become so embedded into practice it is now considered the standard care, yet for others, there has been a significant decline in compliance to ERAS protocols since the end of the national programme [7]. Following the programme, there has been no on-going formal national programme to support ERAS adoption, and so the effect of ERAS protocols on influencing outcomes at a national level is questionable.

Recent research has highlighted that the programme had no discernible independent effect on decreasing LOS nationally for both THA and TKA [6]. Despite the scientific evidence for ERAS, there is still a knowing–doing gap, and widespread implementation within the NHS has not occurred. Mean LOS remains over 4 days after THA and TKA compared with 2 days in large epidemiological studies in equivalent socialised health care systems [8,9]. It is important that the status of nationwide implementation is highlighted and addressed, because improving surgical outcomes for THA and TKA patients is of critical importance to the NHS. Given the current economic challenges within the NHS, the relative high volume of procedures performed compared to other surgeries (THA and TKA are the most common orthopaedic procedures in the United Kingdom [10]) means that a reduction in LOS for these patients could deliver significant capacity savings to the NHS. Given the homogeneity of the procedure and relative fitness of patients compared to other surgical procedures, it may also be argued that THA and TKA are procedures where pathway improvements should be easier to deliver.

## 3. Why Has ERAS Not been More Widely Adopted within the NHS?

Thus, why is clinical practice not reflecting evidence-based surgical care? When the motives for doing so, namely improved patient outcomes and economic savings are so attractive and needed. The question of ERAS implementation has attracted previous attention [11] and remains unresolved. It is not because the implementation of ERAS for THA and TKA in the NHS is not feasible. Pockets of excellence exist [12,13] and a high-quality service should be possible within all NHS hospitals.

The failure of widespread and complete adoption is multi-faceted, and there are contextual factors, similar to other QI interventions, that may limit the success of implementing ERAS. Whilst some staff may feel positive about the implementation of ERAS [14], previously identified and general barriers to implementing ERAS pathways have been reported to include frontline clinicians being resistant to change, not having enough resources for implementation; difficulties with collaboration and communication across the multidisciplinary team; and local or contextual factors, such as patient complexity or hospital location [14,15]. Conversely, facilitating factors in successful implementation sites are reported to be (1) adapting the programme to fit local contexts, (2) achieving and demonstrating early success, (3) gaining support from both clinicians and hospital leadership, (4) having a strong multidisciplinary ERAS team that regularly communicates and (5) recruitment of supporters and full time ERAS staff or champions [14,15].

These factors resonate with the wider quality improvement literature where context has been found to be a crucial determinant of whether quality improvement projects are successful. Kaplan et al. [16] concluded that strong clinical and managerial leadership at all levels, a supportive organisational culture with high staff motivation for change, the use of process and outcome data to monitor changes, and the use of a recognised QI method (such as a plan–do–study–act cycle) when introducing a QI intervention were all crucial to success.

## 4. Recommendations for the Future Implementation of ERAS

We must refocus our efforts and remember that even though ERAS has been shown to improve clinical outcomes, implementing ERAS itself is not the goal, but instead is an intervention by which patient care can be improved. Instead, it should be recognised that improving a clinical outcome is achieved by combining clinical decisions informed by evidence-based medicine (such as an ERAS protocol) with the needed process or system changes, that allow the right things to be delivered in the right way [17]. Understanding this concept is crucial if we are to understand that “wanting to improve is not the same as knowing how to do it” [18].

The need for perioperative care teams to increase their knowledge of QI approaches is therefore required, and this should include the understanding that QI approaches may involve both QI methods (including techniques such as plan–do–study–act cycles, Lean, and Six Sigma) and QI interventions (such as checklists, care-bundles, and clinical pathways) [19]. This nuance is important because an ERAS protocol should be classified as a QI intervention, and this has not previously been emphasised in the ERAS literature. ERAS protocols are QI interventions intended to improve a process, and the evidence for an ERAS protocol for THA and TKA is well established [4]. In the right context and environment, there is clear evidence for successful deployment and adaption. For example, outpatient surgery for THA and TKA is now possible when implementing ERAS informed peri-operative protocols [20]. However, as highlighted previously, the successful deployment of ERAS protocols across all hospitals has not been universal because of contextual factors, and the relationship between reduced compliance of ERAS components to poorer outcomes has been shown [21].

This is important because one of the key contextual factors identified by Kaplan et al. [16] to be associated with successful quality improvement efforts, that has received minimal attention to date within the ERAS literature, is the use of a specific QI method (such as plan–do–study–act cycle, Lean, and Six Sigma) when introducing an ERAS protocol to a specific hospital. A QI method is defined as a “systematic technique for identifying defects in clinical systems and making improvements, typically by involving process measurement and remeasurement” [19]. As such, it may be considered a vital factor in the successful adaptation and implementation of ERAS protocols in varying settings and contexts. This is alongside the more widely described and acknowledged factors such as clinical and managerial leadership, the role of an ERAS champion, a supportive organisational culture, effective multidisciplinary communication and collaboration, and the use of data and ongoing audit [22].

## 5. Conclusions

Implementing an ERAS protocol involves the introduction of a QI intervention into a dynamic environment, across multiple departments, with a varied network of multidisciplinary relationships, and it normally challenges existing working traditions. With such a complexity of factors and variables, it is extremely difficult to introduce an ERAS protocol without the use of a QI method to help understand current processes. It is therefore recommended that to improve the success of implementation, perioperative care teams must understand the role of utilising a QI method to adapt and implement ERAS protocols to their specific context. The future use and evaluation of the use of QI methods to implement ERAS should be encouraged, so that perioperative teams can transition from a will to improve, to an understanding of how to improve.
